# Machine learning of time series data using persistent homology

**DOI:** 10.1038/s41598-025-06551-3

**Published:** 2025-07-01

**Authors:** Takashi Ichinomiya

**Affiliations:** 1 Gifu University School of Medicine, Yanagido 1-1, Gifu, 501-1194 Japan; 2 The United Graduate School of Drug Discovery and Medical Information Science, Gifu University, Yanagido 1-1, Gifu, 501-1194 Japan

**Keywords:** Topologica data analysis, Time series analysis, Persistent homology, Recurrence plot, Applied mathematics, Nonlinear phenomena

## Abstract

This study proposes a novel method for time-series analysis based on persistent homology. Traditional time-series analysis techniques based on persistent homology often involve high computational costs. To address this challenge, we introduce the use of recurrence plots. In our approach, recurrence plots are first generated from the datasets, and topological information is then extracted from these plots using persistent homology. The obtained topological information are vectorized through persistence image and the resulting vectors are further reduced using non-negative matrix factorization. The features derived from this method encapsulate rich and distinctive information inherent in the dataset. We applied the proposed approach to several synthetic and experimental datasets to demonstrate its effectiveness. Our method successfully identified the periodic-to-chaotic and chaotic-to-chaotic transitions in Chua’s system and revealed distinguishing characteristics in electromyograms from healthy, neuropathic, and myopathic individuals. Additionally, the extracted features enabled accurate classification of electrocardiogram data. Overall, the results indicate that the features obtained through this method capture essential information embedded in time-series data.

## Introduction

Topological data analysis (TDA) is an emerging method for analyzing large and complex datasets^1^. In TDA, we focus on topological properties such as the number of connected components, loops, and cavities. Using these properties, we can characterize the nontrivial structures embedded in the dataset.

Persistent homology(PH) is one of the most popular methods in TDA^2^. In PH, we consider a filtered complex - a sequence of complexes $S_1, S_2, \dots , S_N$ that satisfies $S_i \subseteq S_j$ for $i \le j$. Filtered complexes commonly appear in various types of datasets. For example, a grayscale image can be represented as an integer matrix, where each element corresponds to the brightness of a pixel. If we define $S_i$ as the complex comprising pixels whose brightness values are less than *i*, we can construct a filtered complex. In the case of point cloud data, several methods are used for creating filtered complexes, including Vietoris-Rips filtration and Čech filtration. Using PH, we obtain topological properties represented by a set of points in 2D space, which can be visualized using persistence barcodes or persistence diagrams(PD). PH can be applied to a wide variety of data and is used in many scientific fields, including physics^3^, chemistry^4^, medicine^5^, and the social sciences^6^.

While PH is a powerful and unique tool for dataset analysis, it is still in its infancy, and several inherent limitations remains. For example, the output of PH is not directly suitable for machine learning(ML) methods, which includes support vector machines, principal component analysis (PCA), and deep learning. Most ML techniques require input data in the form of fixed-length vectors , whereas PH results in a multiset of points in two-dimensional space. Therefore, to use ML, it is usually necessary to convert the output of PH into a vector format. Several vectorization methods such as persistence landscape^7^ and persistence images(PI)^8^ have been proposed. Another challenge is the difficulty in constructing filtered complexes for certain data types. For example, it is not straightforward to define a filtration for a set of complex numbers. Another important example is color images, where each pixel’s color is represented by three values. In such cases, introducing a filtration is not simple.

Time-series data also presents the challenges for filtration. Generally, time-series data are given as a sequence of vectors ${\varvec{x}}_i$, $1\le i \le T$. Several methods have been proposed for applying PH to time-series data^9^. A standard method is based on the sliding window approach^10^. Using this, we construct a point cloud from the time-series data via delay embedding and then apply PH. Delay-embedded trajectories reflect the structure of attractors^11^; hence, their topological properties reflect those of the attractors. Because this method captures the shape of the trajectory, it is robust to the variations in speed. It has shown excellent performance in identifying periodicity in gene expression and has been applied to various time-series datasets, including financial data^12^. However, this method also has limitations. It is difficult to analyze complex time-series patterns, such as chaotic dynamics, using this approach. Another important drawback lies in the dimension of the embedded space. The computational cost of PH analysis is approximately by $O(N^{D/2})$, where *N* is the number of points in the point cloud and *D* is the embedding dimension^13^. Therefore, as the embedding dimension increases, the computational cost increases exponentially, making it impractical to apply this method to high-dimensional systems. Another popular method is based on sublevel filtration. For univariate time-series data *x*(*t*), we consider the sublevel set $s(r) = \{t \vert x(t) < r\}$ and construct a filtered complex based on *s*(*r*). This approach has been applied to analyze various types of time-series data, such as electroencephalograms^14^ and heart rate variability^15^. Compared with point cloud-based methods, this approach has lower computational cost; however, the relationship between the PH results and the structure of the attractor remains unclear.

We recently proposed a new approach for time-series analysis using PH^16^. The key idea is to use recurrence plots^17^, which are widely used technique in nonlinear time-series analysis. In this approach, we first performed delay embedding and compute the distance matrix *D*, where $D_{ij}$ is the distance between the system states at times *i* and *j*. Taking $D_{ij}$ as a grayscale image, we apply PH analysis. The computational cost for this method remains low even when the embedding dimension is large , and is roughly $O(N^2)$, where *N* is the number of the samples, because PH is applied to $N^2$ points in a two-dimensional space. Thus, this approach is computationally more efficient than the point cloud-based method when the embedding dimension is high.

In a previous paper, we demonstrated our method using simple examples such as the logistic map and the Rössler attractor, showing that PH can qualitatively distinguish between chaotic and periodic behaviour. In this paper, we extend that work by showing how PH can be used to extract features from more complex time-series datasets using PI and non-negative matrix factorization (NMF)^18^.

The remainder of this paper is organized as follows. First, we describe our time-series analysis method, which consists of four main steps, briefly introduced below. The Results section presents the outcome of our analysis. Our method effectively addresses several tasks, such as identifying transitions between chaotic and periodic motion, clustering electromyograms(EMGs), and classifying electrocardiograms(ECGs). Finally, before concluding the paper, we discuss the results and highlight some unresolved challenges.

## Methods

**Fig. 1:**
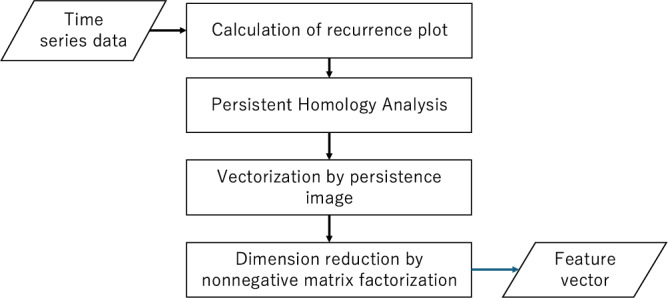
Flow chart of our method.

The proposed method comprises four steps as shown in Fig. 1 . First, we constructed the recurrence plot using the delay-embedded time-series dataset. Second, the recurrence plot was analyzed using PH. Third, we vectorized the PD obtained in the second step using PI. Finally, we reduced the dimensions using NMF. In the following subsections, we describe each step. Python 3.11.11, scikit-learn 1.3.2^19^ and homcloud 4.1.0^20^ were used for the analysis.

### Calculation of recurrence plot

We assume that we have time-series data $x_i$, where $x_i$ is a real number, and $1\le i \le T$ is the time index. The first step in the proposed method is to calculate the recurrence plot using delay embedding^11^. In this embedding, we construct a series of *k*-dimensional vectors:

1 $$\begin{aligned} {\varvec{X}}_i = (x_i, x_{i+1},\dots , x_{i+k-1}) \end{aligned}$$

for $i=1, 2,\dots T-k+1$. After embedding, we create a distance matrix *D*, defined by

2 $$\begin{aligned} D_{ij} = d({\varvec{X}}_i, {\varvec{X}}_j), \end{aligned}$$

where $d({\varvec{X}}, {\varvec{Y}})$ denotes the Euclidean distance between ${\varvec{X}}$ and ${\varvec{Y}}$. A recurrence plot is obtained by considering $D_{ij}$ as a two-dimensional image.

In this process, we must determine the dimensions of embedding space. Unfortunately, because there is no rule of thumb to determine *k* we use $k=3$.

### Persistent homology of recurrence plot

A deep knowledge of algebraic topology is required to define PH precisely. In this subsection, we briefly explain the basic concept of this method. For a rigorous definition of PH, see Zomorodian et al.^21^

After calculating *D*, we analyze its topological properties of this matrix using PH based on a cubical complex. In this analysis, we consider the set *S*(*r*) defined by

3 $$\begin{aligned} S(r) = \{(i, j) \vert D_{i,j} \le r \}. \end{aligned}$$

Because *D* is a non-negative matrix, *S*(*r*) is empty for $r< 0$ and $S(r) \subseteq S(r^\prime )$ if $r \le r^\prime$. This property allows us to construct a filtered complex and perform PH analysis.

**Fig. 2:**
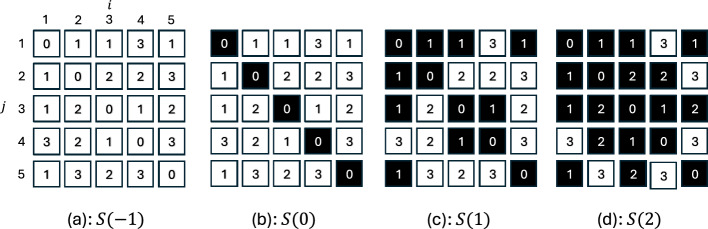
Example of persistent homology.

For example, assume that *D* is as shown in Fig. 2. In this figure, there are $5\times 5$ cells, and each cell has a real number. First, we consider $S(r=-1)$ as shown in Fig.2a. In this case, $S(-1)$ is empty because all the numbers in the cells are larger than $-1$. Next, we consider the case $S(r=0)$ shown in Fig.2b. We find five cells with values of 0. These cells are not in contact with each other, and *S*(0) has five connected components. When we increase *r* to 1, as shown in Fig.2c, we also find five connected components. Fig.2d shows four connected components at $r=2$. Finally, at $r=3$, all the cells are included in *S*(*r*) and there is only one connected component. Therefore, the numbers of connected components are zero, five, five , and four for $r=-1, 0, 1, 2, 3$.

**Fig. 3:**
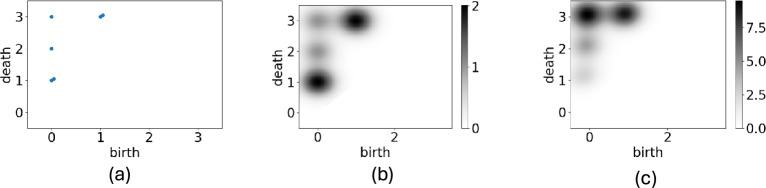
The PD obtained from data shown in Fig. 2. (**a**) Original diagram. (**b**) Smoothed diagrams using a Gaussian kernel. (**c**) Diagram weighted by lifetime.

From the filtered complex, we obtain more information on the topological properties of *S*(*r*). We focus on the cells at $(i,j)=(1,1)$ and (2, 2). At $r=0$, the cells are disconnected. Increasing to $r=1$, we find that these two components are connected by the cells with a value of 1. Therefore, we can conclude at $r=1$, one of these connected components “dies” and is absorbed by the other component. The theory of PH asserts that we can assign two real numbers named “birth” *b* and “death” *d* to every connected component. In the case of Fig.2, five connected components have birth 0. Two of these components die at $r=1$, one dies at $r=2$, and another at $r=3$. We also have two connected components born at $(i,j)=(1,5)$, and (5, 1), whose births and deaths are (1, 3). In PH, we mainly investigate the distribution of (*b*, *d*), which is expressed in a scatter plot named the PD, as shown in Fig. 3a. The values of births and deaths indicate the values at the local minimum and saddles in *D*, respectively.

Here we calrify several important points regarding PH. First, we focused solely on the changes in connected components in this study. Although PH can also characterize more complex topological structures such as loops or voids, our analysis was limited to connected components. Second, there are multiple ways to define “contact” between cells. For instance, in Fig. 2b, there are five connected components if we consider only cells that share an edge as being connected. However, one could also assume that the cells are connected if they touch at a corner. The former definition is known as the V-construction, while the latter is referred to as the T-construction^22^. In this study, we adopted the V-construction for the PH analysis. Third, minor noise in the dataset tends to generate many points near the diagonal line $b=d$ in the PD. According to the stability theorem, small perturbation in the input data do not produce points with long lifetimes, i. e., points with a large $d-b$, while it can produce points with short lifetimes. Therefore, feature engineering is typically employed to reduce the influence of these short-lifetime points. The details of the feature engineering process is described in the following subsection.

### Vectorization of recurrence PH

Using PH, we obtain a multiset of two-dimensional points $\{(b_i, d_i)\}$. However, applying machine learning to such a multiset of points is difficult. Many machine learning algorithms, such as PCA, support vector machines, and deep learning, assume that the size of the input is fixed. In PH, the number of points in the PD is not fixed, and it is impossible to determine this size before the PH analysis. Therefore, this multiset must be converted into a fixed-dimensional vector. Additionally, as discussed in the previous subsection, it is necessary to reduce the influence of points with short lifetimes.

PI is a popular method for vectorizing the results of a PH analysis^8^. Intuitively, the PI is given by a two-dimensional image with the point density in the PD and weights determined by the lifetime. The persistence image *PI*(*b*, *d*) is defined as

$$\begin{aligned} PI(b, d) = w(b,d)\sum _i K(b_i-b, d_i-d), \end{aligned}$$

where, respectively, *w*(*x*, *y*) and *K*(*x*, *y*) are the non-negative weight and kernel functions. To understand the PI, we consider the PD shown in Fig. 3a. The first step to obtain PI is replacing the points in the PD with “clouds” defined by the kernel *K*(*x*, *y*). Using the Gaussian distribution function as *K*(*b*, *d*), we obtained the density plot shown in Fig,3b. The second step involves multiplying these values by a weight function *w*(*x*, *y*). After this process we obtained the weighted smoothed PD, as shown in Fig.3c.

To calculate the PI, we must define the kernel function *K*(*x*, *y*) and the weight function *w*(*x*, *y*). For the kernel function, the Gaussian distribution function $g(x,y) =\frac{1}{\sqrt{2\pi \sigma ^2}}\exp \left( -\frac{x^2+y^2}{2\sigma ^2}\right)$ is typically used, and was employed here. However, there is no general rule for selecting *w*(*x*, *t*). This study used the square of the lifetime as the weight function, $w(x,y) = (y-x)^2$.

### Non-negative matrix factorization of persistence image

Following vectorization using PI, the dimensions of the vectors are reduced. One of the standard methods for dimensional reduction is PCA. However, PCA often gives results that are difficult to interpret. This study used NMF for dimension reduction^18^.

Suppose that there are *N* samples with non-negative *D*-dimensional feature vectors ${\varvec{v}}_i$ $(1\le i \le N)$. In NMF, we reduced the dimension *D* into $D^\prime$ by approximating *V* as

4 $$\begin{aligned} V \sim P Q \end{aligned}$$

where *V* is $N\times D$ matrix $V=[v_1, v_2,\cdots v_N]^T$, and respectively *P* and *Q* are $N\times D^\prime$ and $D^\prime \times D$ non-negative matrices. This approximation is obtained by minimizing $\vert \vert V-PQ \vert \vert$, where $\vert \vert X\vert \vert$ represents the Fröbenius norm of *X*. Each row of *P* represents the reduced expression of the corresponding sample, and each column of *Q* represents the basis used in the reduction.

An advantage of NMF is that it captures the local information within the data. Although there is no theoretical proof, empirical results such as those involving facial images have shown that NMF tends to produce strongly localized basis components^23^. Compared with PCA, this property often makes the results of NMF more interpretable.

NMF also has several disadvantages compared to PCA. First, there is no clear method for determining the rank $D^\prime$. Several methods for rank selection have been proposed, such as those based on cophenetic correlations^24^, the Akaike information criterion^25^, and Bayesian inference^26^. However, these methods have a high computational cost, and currently, the best method is to use expert knowledge. We performed NMF for several $D^\prime$ values and confirmed that the results were qualitatively consistent. We also note that computing NMF requires more processing resources than PCA. This cost is negligible if the number of samples is $O(10^3)$; however, it is important when investigating larger data.

## Results

### Attractors of Chua’s oscillator

First, we present the results of analyzing Chua’s oscillator, which is described as follows:

5 $$\begin{aligned} \dot{x}&= \alpha (y-h(x)) \end{aligned}$$

6 $$\begin{aligned} \dot{y}&= x-y + z \end{aligned}$$

7 $$\begin{aligned} \dot{z}&= \beta y, \end{aligned}$$

where $h(x) = m_1 x +\frac{1}{2}(m_0-m_1)(\vert x+1 \vert -\vert x-1\vert )$. We set $\alpha = 15.6$, $m_0 = -\frac{8}{7}$, $m_1 = -\frac{5}{7}$ and changed $\beta$ from 30.0 to 32.0.

We used *x*(*t*) for $t=1,2,\dots , 1000$ as the input data. We created embedded vector ${\varvec{X}}(t) = (x(t), x(t+1), x(t+2))$ from these data, calculated the distance matrix, and performed the PH analysis. Using NMF, the PIs obtained for each $\beta$ were reduced into a four-dimensional space.

**Fig. 4:**
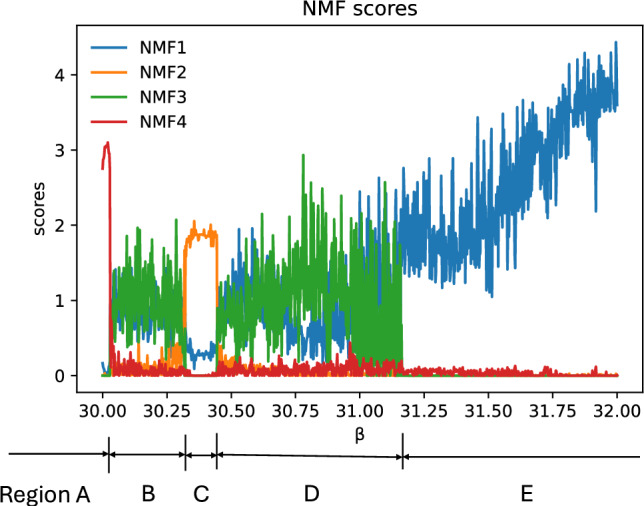
NMF decomposition of Chua model. Results: The blue, orange, green and red lines show the 1st, 2nd, 3rd and 4th NMF scores.

Figure 4 shows the NMF components for $30.0 \le \beta \le 32.0$. This figure clearly shows that there are at least five phases. In Region A, at $\beta \sim 30.0$, the 4th NMF component has large score, while the other components remain small. As $\beta$ increases, this component suddenly decreases to zero in Region B, while the 1st and 3rd components rise sharply. This phase is maintained until $\beta \sim 30.3$, when both components disappear. In Region C, $30.3 \lesssim \beta \lesssim 30.5$, the 2nd NMF component’s score is large, whereas the other components remain small. This phase breaks at $\beta \sim 30.45$, where the 1st and 3rd components rise sharply. Region D is changed to Region E at $\beta \sim 31.1$, where the 3rd component disappears, and only the 1st component survives at $\beta \gtrsim 31.1$.

**Fig. 5:**
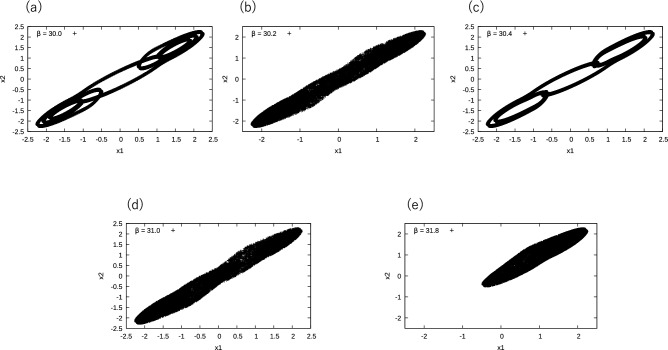
Example: Trajectories of Chua’s system for several $\beta$. (**a**) $\beta =30.0$, (**b**) $\beta =30.2$, (**c**) $\beta =30.4$, (**d**) $\beta =31.0$ and (**e**) $\beta =31.8$.

These five regions correspond to the periodic and chaotic regions of the system. In Fig.5, we show the plot of $(x_1, x_2)=(x(t), x(t+1))$ for several $\beta$. This figure shows that, in Regions A and C, the dynamics are periodic, whereas in Regions B, D, and E they are chaotic. The PH provides more detailed information on these phases. First, we compare the periodic orbits shown in Fig.5a, c. It was difficult to determine whether these two phases were qualitatively distinct. However, the NMF scores represented in Fig. 4 clearly show the qualitative difference between them. In the case of the chaotic phases, there was no clear difference between Regions B and D, whereas Region E showed a distinct difference. It is known that, in Region E, the system possesses two chaotic attractors with separate basins of attraction. The trajectory in Fig. 5e shows only one of these attractors, and two attractors are merged for smaller $\beta$. The results of the PH analysis were consistent with this claim. In Region E, only the 1st NMF component has a large value. This suggests that this component represents the chaotic motion as described in Fig. 5e. In Regions B and D, a new component NMF3 appeared, while NMF1 still remained large. This suggests that NMF3 represents the chaotic motion between the two attractors in Region E.

**Fig. 6:**
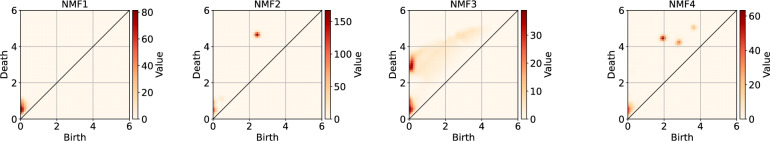
Basis obtained by PH and NMF for Chua’s system.

The basis of NMF is shown in Fig. 6, which provides more detailed information on the phase transition of this system. From this figure, we note that the bases of NMF2 and NMF4 have sharp peaks, while those of NMF1 and 3 have broad ones. This observation supports our suggestion that NMF2 and NMF4 correspond to periodic motion, whereas NMF1 and NMF3 capture the chaotic trajectories. For periodic trajectories, the distance matrix $D_{ij}$ is periodic and the obtained PD comprises several large peaks^16^. The number of spots on the NMF2 basis was smaller than that on NFM4, which indicates the trajectory corresponding to NMF2 was simpler than that corresponding to NMF4. This is consistent with the result shown in Fig.5a, c. In the case of the bases for the chaotic orbits NMF1 and NMF3, the positions of the peaks at death are $\sim 3.0$ in the case of NMF3. This large death implies that the trajectory corresponding to NMF3 spread widely in phase space. This was consistent with the trajectories shown in Fig. 5b, d, e.

### Classification of electromyograms

The second example is the analysis of EMG obtained from patients with neuropathy and myopathy. EMG is a record of the electrical activity in muscles and is widely used to diagnose neuromuscular diseases. Neuropathy and myopathy are neuromuscular diseases associated with muscle dysfunction. In neuropathy, the muscles are normal; however, the nerves that send signals to the muscles are damaged. In myopathy, the nerves are normal, whereas the muscles are dysfunctional. The differences between these two diseases can be captured using EMG.

**Fig. 7:**
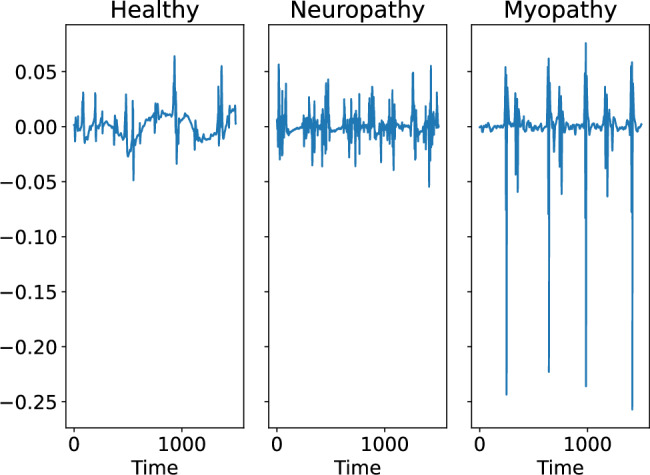
Examples of electromyogram signals. Left: healthy person, middle: neuropathic person, right: myopathic person.

We investigated the EMG dataset provided by the UEA multivariate time series classification archive^27^. This dataset was created by splitting the 4 kHz EMG data published in PhysioNet^28^. The dataset was classified into three classes; signals from healthy person, myopathic person and neuropathic person. This dataset includes 141 samples and each sample is given by a vector with a length of 1500. Fig.7 show the examples of signals obtained from healthy, neuropathic and myopathic persons. We created the embedding vector $X_i=(x_i, x_{i+1}, x_{i+2})$ from each time series $x_i (1 \le i \le 1500)$ and analyzed it using PH and NMF and mapped the dataset onto a two-dimensional space.

**Fig. 8:**
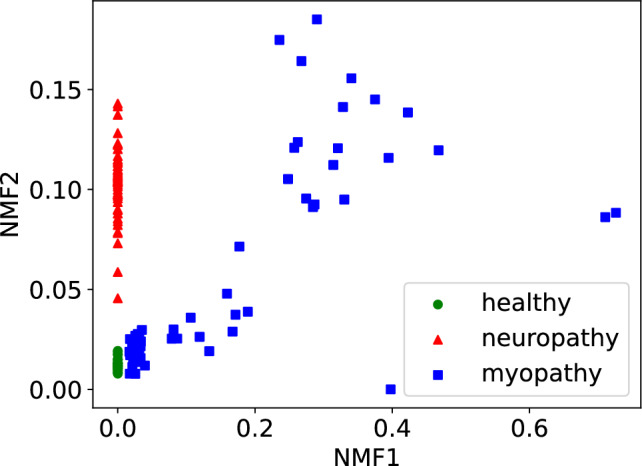
Scatter plot of reduced EMG data. Green circles, red triangles and blue squares respectively represent samples from healthy, neuropathic and myopathic subjects.

Figure 8 shows the result of dimension reduction. In this figure, the green circles, red triangles, and blue squares respectively represent the data from healthy, neuropathic, and myopathic subjects. This demonstrates that our method can effectively distinguish among the three groups. First, data from the healthy subjects exhibit small values for both the first and second components. In the case of neuropathy, the first component remains small, while the second component becomes large. Conversely, myopathy is characterized by a large first component.

**Fig. 9:**
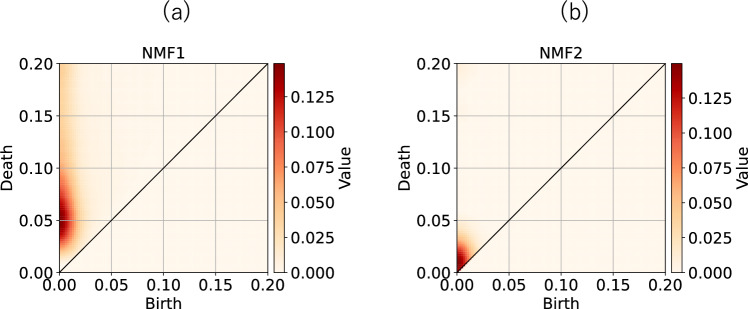
Basis of NMF obtained from the PH analysis of EMG data. (**a**) 1st and (**b**) 2nd components.

To investigate the properties captured by NMF, we show the basis for NMF in Fig. 9. Notably the basis shown in Fig. 9a has a large broad peak at $(b,d) \sim (0, 0.05)$. This implies that if there are many points in this region, the first NMF score enlarges. Figure 9b suggests that if there are many points around $(b,d)\sim (0,0)$ in PD, the 2nd NMF score enlarges. From these analyses, we conclude that the difference between the PDs of healthy subjects and neuropathic subjects arises from points with small births and deaths. Conversely, the PD of myopathic subjects has many points with small births and large deaths.

### Classification of ECG5000 dataset

**Fig. 10:**
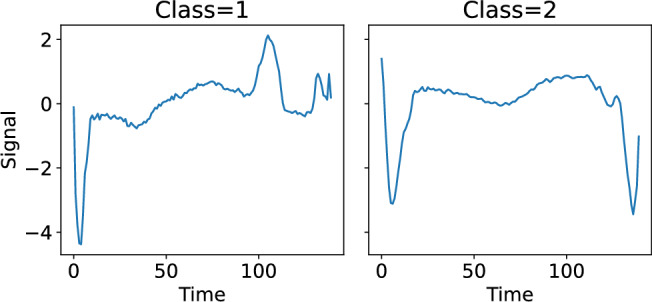
Examples of electrocardiograms in ECG5000 dataset. Left: class = 1, right: class = 2.

The last example is ECG data. In this example, we investigated the ECG5000 dataset. This data set, provided by the UCR time series classification arxiv^29^, was created from the 20 hours ECG signal from one person^28,30^. Each sample in this dataset, the length of which is 140, represents one heartbeat. The dataset comprises 500 training samples and 4,500 test samples classified into five classes. Notably, there is a strong imbalance in the samples with approximately 90 % belonging to classes 1 or 2. In this study, we removed samples that belonged to other classes and only considered classes 1 and 2. Examples of ECGs for classes 1 and 2 are given in Fig. 10. We embedded each time series into a three-dimensional space, calculated the recurrence plot, and performed a PH analysis. The PH result was reduced to a two-dimensional space using NMF.

**Fig. 11:**
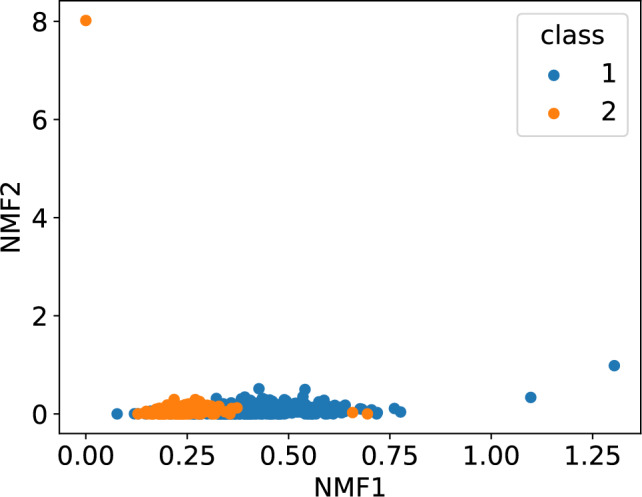
Scatter plot of the reduced ECG5000 training data. The blue and orange circles respectively represent the data of Classes 1 and 2.

**Fig. 12:**
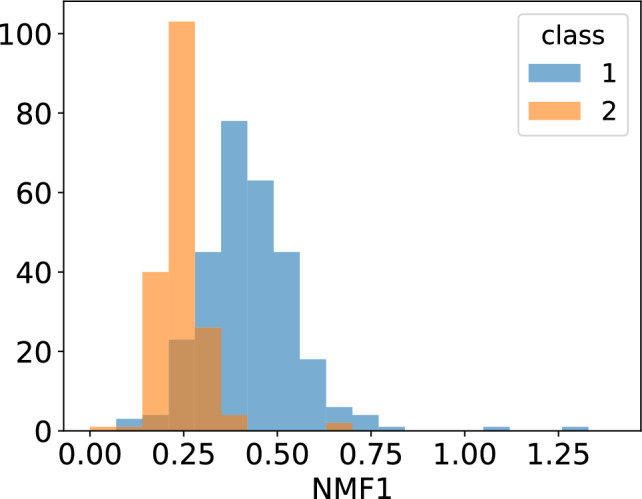
Histogram of NMF1 scores of the ECG5000 training data. The blue and orange histograms respectively show the distribution of Class 1 and 2 data.

**Fig. 13:**
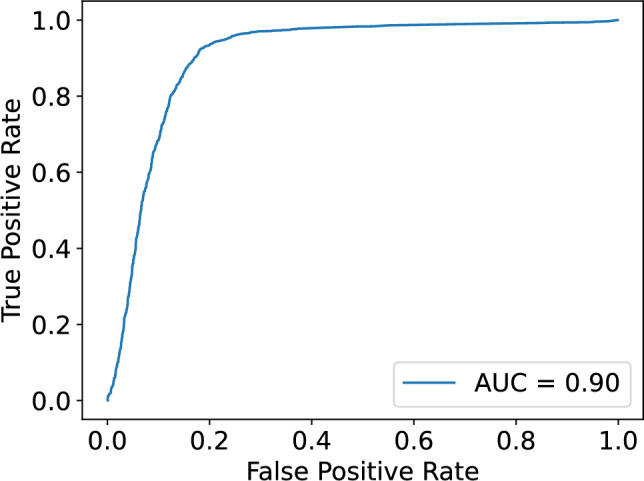
ROC curve when using NMF1 score as the classification variable. *AUC* area under the curve.

**Fig. 14:**
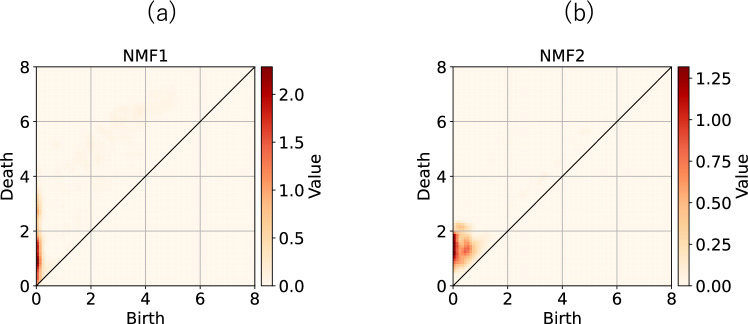
The basis corresponding to NMF1 and NMF2 obtained from the PH analysis of the ECG5000 training dataset.

Figure 11 shows the scatter plot obtained from the 500 sample training dataset in ECG5000. Clearly, the distribution of the first NMF component differs between classes 1 and 2. To investigate the differences in distributions, we created a histogram of NMF1 for each class, as shown in Fig. 12. The distribution of Class 1 had a broad peak at NMF1$\sim 0.3$, whereas that of Class 2 had a sharp peak at NMF1$\sim 0.2$. Regarding NMF2, only one sample had a large score, suggesting that NMF2 captures the characteristics of this outlier. This result indicated that NMF1 scores can be used for classifying ECGs. To check the NMF1 classification performance, we calculated the ROC curve. Using the basis obtained from the training data, we calculated the NMF1 score for test samples and obtained the ROC curve. The results are shown in Fig.13. The area under the curve was 0.90, implying that NMF1 has a good characteristics for this classification. The basis shown in Fig. 14 shows that the NMF1 strongly depends on the points of small births and large deaths, which affords insight into the difference between the two classes of ECG. Regarding NMF2, the basis of NMF2 has a broad peak at (birth, death) $\sim (0, 1.5)$.

For comparison with the state-of-the-art method, we calculated our method’s optimal accuracy. Using NMF1 for the classification, the accuracy was 62%. Conversely, deep-learning algorithms provide accuracy of> 95%^31^. Therefore, the main advantage of our method lies in its explainability rather than its accuracy. Understanding the rationale behind decisions made by deep learning models is often challenging; in contrast, our approach offers interpretability through the basis components derived from NMF. It is also worth noting that the classification performance can be further improved by incorporating additional NMF components and employing more sophisticated classification algorithms. For example, the kernel support vector machine using both NMF1 and NMF2 scores produced an accuracy 87.0 %.

## Discussion

The results showed that our method enables us to extract the nontrivial essential features embedded in the dataset and will be applicable to other data such as financial and meteorological datasets. This method require less computational cost than point-cloud based method. In recent years, there have been several attempts to analyze meteorological or economic data using persistent homology^12,32^. Our method will be applicable to these types of datasets. Compared to other PH-based time-series analysis methods, our approach has the advantage of avoiding the increased computational cost associated with embedding into high-dimensional spaces. As mentioned, it is usually impossible to conduct a PH analysis in high dimensional space. In contrast, our method has no limitation on the dimensionality of the embedding space, because we conducted PH analysis for a two-dimensional image generated by a recurrence plot.

Unfortunately, several limitations remain. First, we must define the distance function before analysis, which is often challenging for multivariate time-series datasets. In the case of the univariate time-series data used in this study, we intuitively employed the Euclidean distance to construct the distance matrix. However, for multidimensional data, there is no inherent distance measure, even though defining a distance is crucial for the analysis. In some cases, well-known distances such as the Mahalanobis distance may work well, but in real-world problems, we often need to define a custom distance. For instance, when analyzing a 12-lead ECG dataset, the input data consists of twelve-dimensional vectors. These twelve variables are nonlinearly and strongly correlated with each other, possibly with some delays. In that case, we must define the distance suitable for our purpose, which is a difficult task. Second, computational cost increases when dataset length increases. As we have noticed in Introduction, the computational cost for PH analysis is roughly proportional to the square of sample size. This cost is smaller than the one of the point-cloud based analysis for large embedding dimension. However, if time-series data’s length is > $10^5$, the computational cost for PH becomes too large. Third, our method cannot detect the changes in frequency. For example, consider the case of distinguishing between homoclinic and periodic orbits. In both cases, the trajectory appears circular. However, for the homoclinic orbit, the period of oscillation increases over time, while for the periodic orbit, the period remains constant. Our method cannot distinguish these different types of orbits because it does not explicitly incorporate time information.

Some of these limitations could be addressed by improving our methods. For multivariate datasets, we could use the feature vectors obtained from the PH of each variable as inputs to another machine learning algorithm, such as deep learning. In this approach, the challenge of distance definition could be resolved by deep learning models. To our knowledge, no previous study has successfully constructed neural networks capable of generating the corresponding PD. This suggests that PH captures information that deep learning models might miss, and thus, features obtained from PDs could provide unique characteristics to enhance deep learning performance. For the detection of frequency changes, PH based on erosion distance will be available^33^. To explain the erosion distance, we consider the case that the two-dimensional bitmap image is provided. In this case we can construct the filtration using the distance from the “boundary”, i.e., the position where white and black cells meet. This type of PH can be used to analyze recurrence plots by introducing $D_{ij}=\theta (a-d({\varvec{X}}_i, {\varvec{X}}_j) )$, where $\theta (x)$ is the Heavyside function and *a* is a threshold. PH based on erosion distance can detect the difference between periodic and homoclinic orbits, because erosion distance explicitly depends on time. The difficulty of this approach is that the result of the PH strongly depends on the threshold *a*. To overcome this difficulty, the promising approach of using multidimensional PH^34^ will be tested, in which the filtration is performed simultaneously using a threshold and erosion distance.

## Conclusion

This study proposes a PH analysis method for time-series data, with a key focus on applying PH to recurrence plots derived from delay embedding. Compared to other PH-based time-series analysis methods, this approach offers a lower computational cost for large embedding dimensions. The PH analysis provides valuable insights into the system through PI vectorization and NMF-based dimensionality reduction. We demonstrated the method’s effectiveness by using both artificial and real-world data. These results suggest that the proposed method can extract essential features from nonlinear time-series data.

## Data Availability

All source codes and dataset used in this study are available from the corresponding author on reasonable request.
